# Evaluation of the Toxicity of Microcyclamide Produced by *Microcystis aeruginosa* in *Danio rerio* Embryos

**DOI:** 10.3390/toxics11020128

**Published:** 2023-01-29

**Authors:** Paloma Nathane Nunes de Freitas, Kazumi Kinoshita Teramoto, Alexander Ossanes de Souza, Ernani Pinto

**Affiliations:** 1 Luiz de Queiroz College of Agriculture, University of São Paulo, Piracicaba 13418-900, Brazil; 2 Nuclear Energy in Agriculture Center, University of São Paulo, Piracicaba 13416-000, Brazil; 3 School of Pharmaceutical Sciences, University of São Paulo, São Paulo 05508-000, Brazil

**Keywords:** cyanobacteria, cyanopeptides, microcyclamide, toxicity, zebrafish

## Abstract

The genus of cyanobacteria *Microcystis* is one of the most recurrent in blooms and is associated with the hepatotoxin microcystin production. In addition to cyanotoxins, these bacteria produce a wide range of secondary metabolites with a wide repertoire of activities. The co-occurrence of cyanotoxins and other cyanopeptides during blooming is quite common, and the negative effects are not always limited to one class of toxins, which makes it essential to investigate the toxicity of the other compounds individually. The objective of this study was to isolate the cyanopeptide microcyclamide produced by the strain *Microcystis aeruginosa* LTPNA 08 by liquid chromatography coupled to high-resolution mass spectrometry with a quadrupole-time-of-flight analyzer (LC-HR-QTOF-MS/MS) and to evaluate its acute toxicity in embryos of *Danio rerio* through the Fish Embryo Acute Toxicity (FET) assay. The fraction containing microcyclamide (95% purity) caused lethality in 62% of the embryos after 96 h exposure (50 µg mL^−1^), with evidence of cardiotoxicity (cardiac edema). The calculated LC_50_ value was 42.98 µg mL^−1^ (with a concentration range of 37.79–48.89 µg mL^−1^). The characterization of the secondary metabolites produced by cyanobacteria and the investigation of the toxicity of these compounds individually are essential for the identification of the substances responsible for negative effects on living organisms and on the ecosystem, in addition to assisting in the development of risk management policies.

## 1. Introduction

The process of anthropic eutrophication of aquatic ecosystems, generated mainly by urbanization, industrialization, and agricultural activities [1], in addition to the increase in CO_2_ levels and global warming, promotes the dominance of cyanobacteria over other phytoplankton organisms, causing a phenomenon known as “blooms” [2,3]. Cyanobacterial blooms represent a threat, due to the possible production of cyanotoxins, causing ecotoxicological problems, in addition to affecting human and animal health. Among the species of cyanobacteria associated with this phenomenon, the genus *Microcystis* stands out, due to its recurrent presence in blooms [4] accompanied with the production of microcystins, which are hepatotoxic but also able to cause reproductive and developmental toxicity, nephrotoxicity, cardiotoxicity, neurotoxicity, immunomodulation, endocrine disruption, and death in animals and humans [5].

In addition to cyanotoxins, cyanobacteria produce numerous other secondary metabolites, such as cyanopeptolines, anabaenopeptins, cyclamides, aeruginosines, microgynines, etc. To date, microcystins are the best known and most studied class of cyanopeptides from an ecological and toxicological point of view. However, although microcystins are an ecotoxicological concern, other metabolites can be frequently detected in surface water and in concentrations equal to or greater than that of microcystins, needing to be considered in environmental and human risk assessment. Furthermore, several studies with cyanobacterial extracts reported in the literature, observed toxic effects that could not be explained by the presence of microcystins alone, suggesting that other metabolites also need to be considered in risk assessments, such as cyclamides [6,7,8].

Cyclamides are cyclic hexapeptides characterized by three azole or azoline rings, probably derived from the modification of cysteine and threonine. Microcyclamide was first reported in the cyanobacterial strain *Microcystis aeruginosa* NIES-298 [9]. These cyclic peptides are synthesized by the ribosomal biosynthesis pathway through a set of enzymes related to patelamide biosynthesis [10]. Some cyclamides showed cytotoxicity in a variety of cancer cell lines and in aquatic organisms. As an example, microcyclamide isolated from *M. aeruginosa* NIES 298 exhibited moderate cytotoxic activity against P388 murine leukemia cells [9]. Aerucyclamides A and B were toxic to the freshwater crustacean *Thamnocephalus platyurus*, exhibiting LC_50_ values of 30.5 and 33.8 μM, respectively [11].

Cyclamides were initially proposed as metabolites with antibacterial activity; however, different activities observed for individual cyclamide structures indicated that the antibacterial action may not reflect the true biological function of these cyclic peptides. According to Ziemert et al. [10], the enormous diversity of cyclic hexapeptide variants suggests that cyclamides may be involved in the communication and self-recognition of *Microcystis* ecotypes. Despite their wide occurrence, the ecological function of these compounds remains misunderstood.

Given this context, it is essential to investigate the toxicity of these compounds. Since cyanobacterial blooms are increasingly frequent [12,13], there is a risk of the presence of potentially toxic metabolites in water, which can directly affect the organisms present there and in turn come in direct or indirect contact with, as well as exert harmful effects on human beings. Thus, the aim of this study was to isolate the microcyclamide cyanopeptide produced by the *Microcystis aeruginosa* strain LTPNA 08 by means of liquid chromatography coupled to high-resolution mass spectrometry with a quadrupole-time-of-flight analyzer (LC-HR-QTOF-MS/MS) and to evaluate its acute toxicity in *Danio rerio* embryos.

## 2. Material and Methods

### 2.1. Solvents, Reagents, and Standards

All solvents (ethanol, methanol (MeOH), acetonitrile (ACN), and isopropanol) and other reagents (ammonium formate, sodium formate, formic acid, ammonium hydroxide, sodium chloride, and trifluoroacetic acid) used for chromatography and mass spectrometry were of high-performance liquid chromatography (HPLC) grade. The solvents used for the extraction (ethyl acetate and dichloromethane) were of analytical grade, as were the reagents used to prepare the ASM-1 culture medium and the medium used in the toxicity tests with *D. rerio* (reconstituted water). Ultrapure water was obtained using a Milli-Q^®^ Direct 8 system (Millipore Corporation, Burlington, MA, USA). Dimethol sulfoxide-d6, the hydrochloric acid solution (6 M in H_2_O), and amino acid standards were purchased from Sigma-Aldrich (Merck, Darmstadt, Germany).

### 2.2. Cyanobacterial Strain

The strain *Microcystis aeruginosa* LTPNA 08 was isolated from the Salto Grande Reservoir (Americana, SP, Brazil) [14] and is part of the cyanobacterial culture bank of the Laboratory of Toxins and Natural Products (LTPNA) of the University of São Paulo (USP). The LTPNA 08 strain was selected as the object of study, as in previous studies the presence of microcyclamide was verified, co-eluting together with microcystins and microginines [14].

For biomass production, the strain was cultivated for 30 days in ASM-1 liquid medium [15] under aeration, light intensity of 70 µmol photons m^−2^ s^−1^, 23 ± 2 °C, and a 12:12 h (light/dark) photoperiod. At the end of each cultivation, the cell material was concentrated by centrifugation (10,000× *g*, 10 min, 4 °C, Eppendorf 5804R), and the obtained pellet was frozen at −20 °C and lyophilized (SNL216V, Thermo Electron Corporation, Waltham, MA, USA).

### 2.3. Microcyclamide Extraction for Isolation and Purification

Extraction was performed according to Sanz et al. [16] and Paiva et al. [17]. For the isolation process, the lyophilized material (1 g) was extracted three times with 70% MeOH (1 g/40 mL^−1^) and subjected to an ultrasound probe for 10 min, pulse of 10 s, at an intensity of 30% (Soni Omni Disruptor—Omni International, Kennesaw, GA, USA). The material was centrifuged (8500× *g*, 4 °C, 10 min, Eppendorf 5804R), and the supernatant was collected and submitted to a rotary evaporator system (Model B-491—Büchi, Flawil, Switzerland), with pressure of 83 mBar, rotation of 100 rpm, and water temperature of 30 °C, to remove the solvent from the sample. The extract was reconstituted in 100 mL of 5% MeOH and applied to the solid-phase extraction (SPE) cartridge Sep Pak C18 Vac 6 cc, 500 mg (Waters, Milford, MA, USA), previously conditioned with MeOH (60 mL^−1^), followed by Milli-Q ultrapure water (60 mL^−1^) for sample fractionation. After sample elution, the cartridges were washed with water (60 mL^−1^) and eluted with a gradient of 60% MeOH acidified with 0.1% formic acid (60 mL^−1^) to obtain fractions enriched with the cyanopeptide of interest. The eluates obtained in the pre-fractionation process were concentrated under nitrogen flow and reconstituted in ACN/H_2_O (30/70, *v*/*v*). The samples were filtered through 0.22 µm PVDF syringe filters (Analytic, São Paulo, Brazil) and subjected to chromatographic analysis by LC-HR-QTOF-MS/MS and, for compound isolation, to semi-preparative high-performance liquid chromatography combined with diode array detection (HPLC-DAD).

### 2.4. Isolation and Purification of Microcyclamide

The 60% fraction containing the cyanopeptide of interest was subjected to chromatographic processes, aiming at the isolation of the pure compound. For this purpose, a semi-preparative liquid chromatography system (Prominence, Shimadzu, Japan) was used, consisting of a controller (CBM-20A), a quaternary pump (LC20AT), a degasser (DGU-20AS), an autosampler (SIL-20AC), a diode array detector (SPD-M20A), and a fraction collector (FRC-6A). Different chromatographic columns, mobile phase compositions, and gradients were tested for the isolation of the microcyclamide compound of *m/z* 583. The best separations were achieved by the Luna C18(2) (250 × 4.6 mm, 5 μm) and Synergi MAX-RP (250 × 4.6 mm, 4 μm) columns, using as the mobile phase A) H_2_O + 0.1% HCOOH and B) ACN + 0.1% HCOOH at a flow rate of 1.0 mL/min. The optimized chromatographic method was then transferred to a semi-preparative scale for the isolation and purification of the compound of interest. The separation was carried out on a Luna C18(2), 250 × 10 mm, 5 μm, semi-preparative reversed-phase column (Phenomenex, Torrance, CA, USA), using (A) H_2_O + 0.1% HCOOH as the mobile phase and (B) ACN + 0.1% HCOOH, with a workflow of 3.0 mL min^−1^.

Microcyclamide was monitored at the maximum wavelengths (λmax, 225 nm). Due to the presence of microcystins in the fractions, it was also monitored at λmax of 238 nm. The chromatographic peaks with a UV absorption spectrum characteristic of the microcyclamide peak (λmax, 205 nm) were collected, and the subfractions were dried under nitrogen flow and analyzed by LC-HR-QTOF-MS/MS to verify the isolation success and peak purity. The subfractions were subjected to successive isolation until obtaining pure fractions of the compound. The purified compound was subjected to mass spectrometry analysis (LC-HR-QTOF-MS/MS). The isolation and purification process was carried out until enough material was obtained for the ecotoxicological tests with *D. rerio*. Altogether, 15 g of dry biomass was used to obtain 13.86 mg of microcyclamide.

### 2.5. Detection and Structural Characterization of Microcyclamide Using Mass Spectrometry

The detection, identification, and structural characterization of the cyanopeptide produced by the LTPNA 08 strain were performed by LC-HR-QTOF-MS/MS, according to the methodology described by Sanz et al. [18]. The chromatographic analyses were carried out in a Shimadzu Prominence chromatography system (Shimadzu, Kyoto, Japan) with a diode array detector (SPD-M20A) coupled to a mass spectrometer with a quadrupole-time-of-flight (QTOF) analyzer (MicroTOF-QII, Bruker Daltonics, MA, USA), equipped with an electrospray source (ESI). The equipment was operated in positive ionization mode, with a capillary potential of 3500 V, a nebulizer gas pressure (nitrogen) of 35 psi, and temperature and flow of the drying gas (nitrogen) of 300 °C and 5 mL min^-1^. A solution of 10 mM sodium formate in isopropanol/water (1:1, *v*/*v*) was used as an external calibrant. The instrument was operated in scanning mode (full scan) and in Auto MS/MS mode, in the range from 50 to 2000 *m*/*z*, performing MS/MS experiments on the three most intense ions obtained in MS scan mode. Chromatographic separations were performed on a Luna C18 column (2) (5 µm, 150 × 2.0 mm), protected with a pre-column of the same composition (Phenomenex, Torrance, CA, USA), at a temperature of 35 °C. The eluents used as mobile phase were: (A) H_2_O containing 5 mmol L^−1^ of ammonium formate and 0.1% of HCOOH, and (B) ACN, at a flow rate of 0.2 mL min^−1^.

Data processing was performed using Data Analysis 4.0 software (Bruker Daltonics, Bremen, Germany). The elemental composition, the deviation between the measured mass and the theoretical mass in ppm (error), and the comparison of the theoretical and measured isotopic pattern values of the peak of interest, by means of a score (mSigma), were calculated using Smart Formula Editor. Molecular formula confirmation was based on the widely accepted limits of 5 ppm and 20 mSigma. The characterization of the cyanopeptide was established based on the data of exact mass, elemental composition, adduct formation, charge states, isotopic pattern, and mainly, fragmentation profile (MS2). The interpretation and confirmation of the amino acid sequence obtained after cyanopeptide fragmentation by mass spectrometry were performed following the methodologies proposed by Paizs and Suhai [19] and Cantú et al. [20]. The amino acid residues were determined by calculating the mass difference between a series of successive fragment ions, identifying ions of the -a, -b, and -y series. In addition to the search for neutral losses of CO (−28 u) from a -b ion (-a ion) and neutral losses of H_2_O (−18 u) and NH_3_ (−17 u) molecules from -b and -y, the search for iminium ions in the low mass region of the spectrum could also provide information inherent to the amino acid composition of a peptide.

### 2.6. Evaluation of the Acute Toxicity of Microcyclamide with D. rerio Embryos

The embryo–larval assays with fertilized eggs of *D. rerio* were carried out following the OECD guidelines—Guidelines for the Testing of Chemicals—Test No. 236: Fish Embryo Acute Toxicity (FET) Test [21]. The microcyclamide test solutions were prepared with the compounds isolated from *M. aeruginosa* LTPNA 08. The stock solutions were prepared by resuspension of the dry material in MeOH (0.1%) and ultrapure water, obtaining solutions of 1 mg mL^−1^ and 2 mg mL^−1^, respectively. From the stock solutions, the test solutions were prepared with reconstituted water at concentrations of 1, 5, 10, 25, and 50 µg mL^−1^.

Fertilized eggs were exposed for 96 h to five different concentrations (1, 5, 10, 25, and 50 µg mL^−1^) of microcyclamide. The assay was carried out in a static system, without renewing the test solution, in 24-well polystyrene plates containing 2 mL^−1^ of solution. Each concentration was tested in triplicate, including the positive (2% of ethanol 96%) and negative (reconstituted water) controls and the solvent-containing control (0.1% MeOH). Five embryos were used per well, totaling 120 embryos in all. The embryos were kept in an oven at 26 ± 1 °C for 96 h and monitored every 24 h with the aid of a microscope (Nikon, Tokyo, Japan) for mortality. At each observation, the dead embryos were counted and removed from the wells to maintain the water quality and avoid contamination. The following lethality parameters were evaluated: (1) coagulation of the fertilized eggs, (2) absence of somite formation, (3) absence of tail detachment from the yolk sac, and (4) absence of heartbeat [21]. Embryos hatch between 48 and 72 h, and from then on, malformations of embryos/larvae were also evaluated, indicative of teratogenicity, such as yolk sac edema, pericardium edema, digestive system edema, caudal curvature and lordosis, absence of eye pigmentation, changes in body size, and behavioral changes such as early or late hatching. At the end of the exposure period (96 h), acute toxicity was determined based on the observation of at least one of the lethality parameters, and when possible, the concentration required to produce death in 50% of exposed individuals (LC_50_) was calculated. Cardiotoxicity, more specifically, was evaluated through the adverse effects of the substances tested on the development of the heart, through the observation of cardiac edema and changes in heart rate, in embryos at 96 h. The heart of the *D. rerio* embryo starts beating at approximately 24 h and is visible during the first week of development, which allows a visual analysis of the effects of chemicals on cardiac formation. The surviving larvae were immobilized for photographic recording and subsequent identification of the morphological alterations caused by the substances under study. At the end of the experiment, the larvae were euthanized by an overdose of tricaine methane sulfonate (MS-222).

### 2.7. Statistical Analysis

The data are expressed as mean ± standard deviation (SD). The data were analyzed using analysis of variance (One-way ANOVA), followed by Tukey’s test for multiple comparisons and Dunnett’s test for comparing treatments with controls, considering a significance of 95% (*p* < 0.05). LC_50_ values were calculated using non-linear regression analysis. Graphs and all statistical tests were performed using GraphPad Prism Version 8.0 statistical software.

## 3. Results and Discussion

### 3.1. Isolation and Purification of Microcyclamide

**Figure 1:**
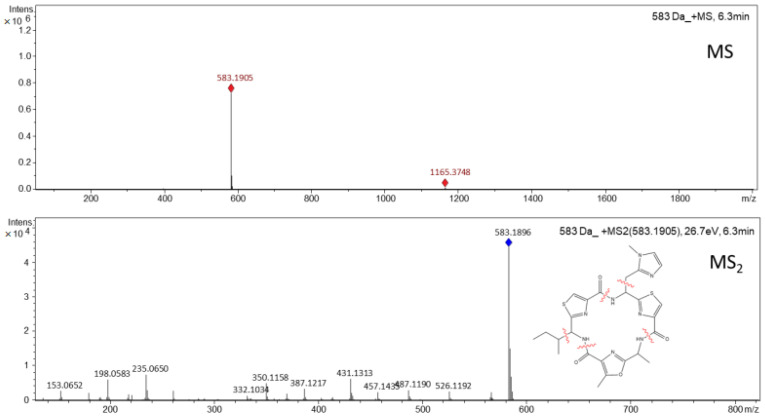
MS and MS_2_ spectra obtained by LC-HR-ESI-QTOF-MS/MS for microcyclamide, *m*/*z* 583.

The isolation and purification step, on a semi-preparative scale, proceeded from the 60% acidified methanolic fractions, obtained in the pre-fractionation process by SPE. A cyanopeptide of molecular mass 582 Da was isolated from the hydroalcoholic extract of *M. aeruginosa* (LTPNA 08) and monitored at the wavelength of 225 nm. The collected subfractions were analyzed again by LC-HR-QTOF-MS/MS until obtaining fractions with compounds with high purity (the microcyclamide purity value was 95%), of molecular ion [M^+^H]^+^ *m*/*z* 583,1896 corresponding to the molecular formula C_26_H_30_N_8_O_4_S_2_ (theoretical *m*/*z* 583.1904, error 1.4 ppm, and mSigma 9.8) (Figure 1).

The experimental and theoretical exact mass and molecular formula were generated by the SmartFormula application of the Data Analysis software, which showed low error values (−0.8 ppm) and mSigma (3.6), indicating high agreement with the proposed molecular formula.

From the MS_2_ spectrum obtained for the substance with molecular mass 582 Da, isolated from *M. aeruginosa* LTPNA 08, and data obtained from the literature, it was possible to verify great similarities with the compounds microcyclamide [9], microcyclamide A [22] and microcyclamide GL582 [22]. The three compounds showed molecular formula and approximate molecular weights similar to those of the compound isolated in this work (microcyclamide: HRFABMS (positive) C_26_H_31_N_8_O_4_S_2_ *m*/*z* 583.1931 [M^+^H]^+^, microcyclamide A: HREIMS C_26_H_30_N_8_O_4_S_2_ *m*/*z* 582.1831 (MH^+^) and microcyclamide GL582: HREIMS C_26_H_30_N_8_O_4_S_2_ *m*/*z* 582.1830 (MH^+^), verified in the analyses performed by high-resolution mass spectrometry.

### 3.2. Evaluation of the Toxicity of Microcyclamide Produced by M. aeruginosa (LTPNA 08) in D. rerio

Microcyclamide embryotoxicity was evaluated through the percentage of mortality and morphological anomalies observed in *D. rerio* embryos and larvae, after 96 h of exposure to different concentrations of this compound. The mortality rates of the negative (reconstituted water) and positive (2% of ethanol 96%) controls and the solvent-containing control (0.1% MeOH) were 3%, 76%, and 100%, respectively.

**Figure 2:**
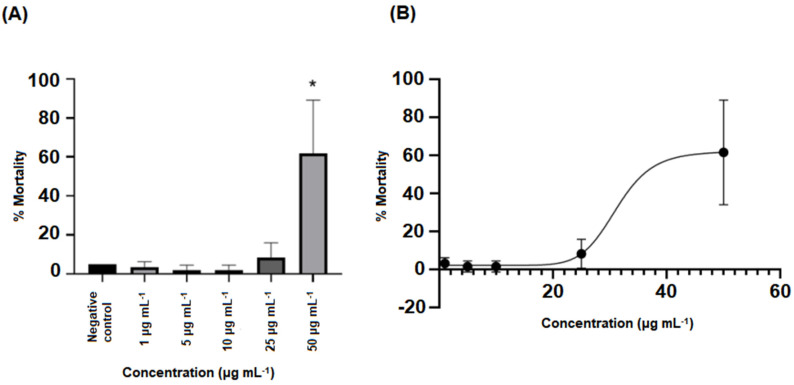
(**A**) Graph of the mortality rate of *D. rerio* embryos exposed to the negative control and to concentrations of 1, 5, 10, 25, and 50 μg mL^−1^ of microcyclamide for 96 h and (**B**) dose–response curve obtained after 96 h of exposure. The error bars indicate the SD of the means (*n* = 3). (*) Dunnett’s test was used, with *p* < 0.05.

Microcyclamide caused mortality in 62% of the embryos at a dose of 50 µg mL^−1^ at the end of the experimental period of 96 h, being this value statistically different (*p* < 0.05) from those recorded for the other treatments and the control (Figure 2). For the other concentrations tested (1 to 25 μg mL^−1^), there were no statistically significant differences (*p* > 0.05) in the lethality rates of embryos/larvae compared to the control.

**Figure 3:**
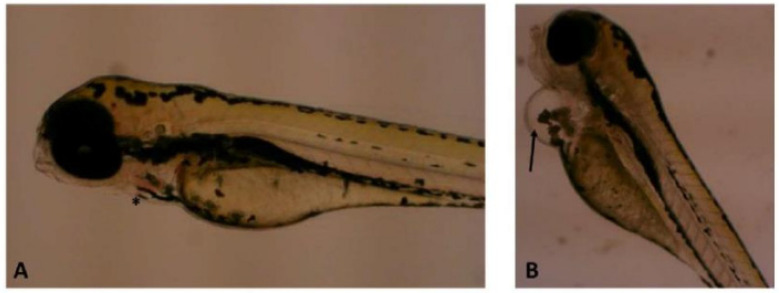
Embryos after 96 h. (**A**) Animal of the negative control group, with normal cardiac development (*) and (**B**) animal exposed to treatment with microcyclamide (50 µg mL^−1^), with edema in the pericardium (→).

From the dose–response curve (Figure 2B), it was possible to calculate an LC_50_ (96 h) for the microcyclamide of 42.98 µg mL^−1^ (with a concentration range of 37.79–48.89 µg mL^−1^). No alterations considered teratogenic were observed up to 96 h at concentrations from 1 to 50 μg mL^−1^, but alterations in cardiac development (pericardial edema) were observed in all larvae treated with 50 μg mL^−1^ of microcyclamide (Figure 3B); with the other concentrations, no alterations in cardiac development (pericardial edema) were observed (1 to 25 μg mL^−1^). Cardiac development (pericardial edema) was visually observed using microscopy (not quantified).

Microcyclamide is commonly produced by cyanobacteria of the genus *Microcystis* and can be detected in the environment at concentrations equivalent to microcystin hepatotoxins. The co-occurrence of these compounds and other cyanopeptides is quite common, both in laboratory-grown cyanobacteria and in the natural environment, which makes the toxicological evaluation of these mixtures extremely complex [6,14,23].

In general, these cyanopeptides are considered non-toxic compounds; however, most of these metabolites have biological activity and are potent protease inhibitors and should not be ignored regarding their toxicological potential. Although a variety of cyanopeptides are simultaneously detected in the environment, the assessment of individual cyanopeptides’ toxicity must be performed to identify which of these metabolites are (eco)toxicologically relevant. An increasing number of cyclic peptides containing heterocyclic amino acids, forming thiazole, oxazole, thiazoline, and oxazoline rings, have been isolated from freshwater cyanobacteria, including nostocyclamide, microcyclamide, and aerucyclamide [9,10,11,22,24,25,26].

Unlike other classes of cyanopeptides, this group of metabolites is synthesized via ribosomal synthesis [10], similar to the patelamide pathway discovered in *Prochloron didemni*, a cyanobacterium that lives in symbiosis with the ascidian *Lissoclinum patella* [27]. In the literature, they are described as metabolites with cytotoxic, antimicrobial, and anti-parasitic properties [9,10,11,22,26,27,28,29]. Microcyclamide was the first cyclic hexapeptide of this class isolated from the cyanobacterium *M. aeruginosa*, showing moderate toxicity against murine leukemia cells (P388), with an IC_50_ value of 1.2 µg mL^−1^ [28]. The anticancer potential of microcyclamides GL616, GL582, GL614A, GL614B, GL614C, GL546A, GL546B, GL628, and microcyclamide A, isolated from a bloom of a *Microcystis* sp., was evaluated in A549 lung and Molt-4 tumor cell lines [22]. Microcyclamide GL582 showed low cytotoxicity in the Molt-4 cell line, with growth inhibition of 20% at a concentration of 10 µg mL^−1^. The other compounds showed no effects on cell lines at the tested concentrations of 1 and 10 µg mL^−1^.

Sadler and Von Elert [29] evaluated the interaction between *M. aeruginosa* and *Daphia magna* and observed a possible defense mechanism induced in *M. aeruginosa* against predation. The presence of *D. magna* stimulated the intracellular production of cyanopeptides (aerucyclamides, cyanopeptolines, and microcyclamide), in addition to microcystin, suggesting a role of these compounds in the protection against herbivory. In addition to increased intracellular production, the presence of *D. magna* induced the release of microcyclamide 7806A into the surrounding environment, which suggests that this extracellular compound may constitute another undescribed line of defense against predation in *M. aeruginosa*.

According to the data in Table 1, it seems reasonable not only to focus on microcystins in order to investigate their toxicity in aquatic organisms such as *Astyanax altiparanae* [8], *Ceriodaphnia dubia*, and *D. magna* [30], but also to take into account other secondary metabolites, which also have supposedly negative effects on the fitness of aquatic organisms.

Cyanopeptolins, for example, act as protease inhibitors and are produced in a non-ribosomal pathway similar to microcystins. Their detrimental effects on zooplankton have been demonstrated by Gademann et al. [31]. The harmful effects of microgynins on *A. altiparanae* [8] and *T. platyurus* [32] and of aeruginosins on *T. platyurus* [33] have also been demonstrated.

Cyclamides constitute a class of cyanobacterial peptides produced by a ribosomal pathway. Little is known about the impact of cyanobacterial cyclamides on zooplankton, but aerucyclamides A and B have been shown to have cytotoxic properties towards the crustacean *T. platyurus*, exhibiting LC_50_ values of 30.5 and 33.8 μM, respectively [11], and the protozoan parasite *P. falciparum* [26].

Cyclamides in general have cytotoxic properties [9] and have been shown to be harmful to crustaceans and protozoa. However, nothing is known about the effects of cyclamides on fish.

The microcyclamide in this study induced a mortality rate above 50% at a concentration of 50 µg mL^−1^. Despite the observed mortality, it can be considered as having low toxicity, as the effects were observed at very high concentrations, which are rarely found in the environment. The cyanopeptide concentrations used in this work, as well as in the work by Ujvárosi et al. [34], were far above the values normally detected in the environment (µg L^−1^) for these compounds, as shown in the study by Beversdorf et al. [35] that quantified the absolute concentrations of cyanopeptides other than microcystins in samples from six eutrophic lakes in the USA. The concentrations observed in that study were in the μg L^−1^ range (i.e., nanomolar). However, evidence of adverse effects of these cyanopeptides at environmentally relevant concentrations indicates that the toxic potential of these compounds should not be ruled out, as they may play a relevant physiological and ecological role, requiring further synergy studies. Cyclamides, for example, have allelopathic activity against other phytoplankton organisms and may be involved in the chemical protection mechanism against herbivory. The toxicological information obtained for these isolated compounds is extremely important and can assist in the development of risk management policies.

## 4. Conclusions

The acute toxicity of microcyclamide was evaluated using the FET assay, which evaluated the toxic effect of this compound on the embryo–larval development of *D. rerio*, an aquatic vertebrate model widely used in ecotoxicology. The compound proved to be slightly toxic to *D. rerio* embryos, causing lethality in 62% of the animals, after 96 h of exposure at a concentration of 50 µg mL^−1^, with evidence of cardiotoxicity (cardiac edema). The calculated LC_50_ value for microcyclamide was 42.98 µg mL^−1^ (with a concentration range of 37.79/48.89 µg mL^−1^). The characterization of secondary metabolites produced by cyanobacteria and the investigation of the toxicity of these compounds individually are essential for identifying the substances responsible for negative effect on living organisms and the ecosystem, which are not limited to the toxins produced. Despite their wide occurrence, the ecological function of these compounds still remains uncertain. The structural complexity of these secondary metabolites produced by cyanobacteria and the available biological data are still not enough to affirm their true function or ecological relevance.

## Figures and Tables

**Table 1:** LC_50_ values of cyclamides (or other secondary metabolites) and cyanobacteria (dry weight).

Organism	Cyanobacteria (Dry Weight) * or Compound	Value of LC_50_	Reference
*Thamnocephalus platyurus*	Cyanopeptolin 1020	8.8 μM	[31]
*A. altiparanae*	*M. panniformis* MIRS-04 (producer of microcystins and micropeptins) *	0.24 mg mL^−1^	[8]
*A. altiparanae*	*M. aeruginosa* NPCD-01 (producer of microginins) *	0.32 mg mL^−1^	[8]
*C. dubia*	Microcystin-LR and [D-Asp^3^]-microcystin-LR	5.53 μg L^−1^	[30]
*D. magna*	Microcystin-LR and [D-Asp^3^]-microcystin-LR	85.72 μg L^−1^	[30]
*Plasmodium falciparum*	Aerucyclamides A, B, C and D)	5.0, 0.7, 2.3 and 6.3 μM, respectively	[26]
*T. platyurus*	*Woronichinia naegeliana* (producer of microginin MG-FR3) *	7.78 μg mL^−1^	[32]
*T. platyurus*	Aeruginosin 828A	22.4 μM	[33]
*D. rerio embryos*	Microcyclamide	42.98 (with a concentration range of 37.79–48.89) µg mL^−1^	[This study]

* Represent Dry Weight of Cyanobacteria.

## Data Availability

Not applicable.
